# Recurrence Patterns After IMRT/VMAT in Head and Neck Cancer

**DOI:** 10.3389/fonc.2021.720052

**Published:** 2021-09-16

**Authors:** Heleen Bollen, Julie van der Veen, Annouschka Laenen, Sandra Nuyts

**Affiliations:** ^1^Laboratory of Experimental Radiotherapy, Department of Oncology, KU Leuven, Leuven, Belgium; ^2^Department of Radiation Oncology, Leuven Cancer Institute, University Hospitals Leuven, Leuven, Belgium; ^3^Leuven Biostatistics and Statistical Bioinformatics Center, KU Leuven, Leuven, Belgium

**Keywords:** radiotherapy, intensity-modulated radiotherapy, volumetric modulated arc therapy, recurrence, head and neck cancer, proton therapy, tumor resistance, delineation

## Abstract

**Purpose:**

Intensity-modulated radiotherapy (IMRT) and volumetric modulated arc therapy (VMAT), two advanced modes of high-precision radiotherapy (RT), have become standard of care in the treatment of head and neck cancer. The development in RT techniques has markedly increased the complexity of target volume definition and accurate treatment delivery. The aim of this study was to indirectly investigate the quality of current TV delineation and RT delivery by analyzing the patterns of treatment failure for head and neck cancer patients in our high-volume RT center.

**Methods:**

Between 2004 and 2014, 385 patients with pharyngeal, laryngeal, and oral cavity tumors were curatively treated with primary RT (IMRT/VMAT). We retrospectively investigated locoregional recurrences (LRR), distant metastases (DM), and overall survival (OS).

**Results:**

Median follow-up was 6.4 years (IQR 4.7–8.3 years) during which time 122 patients (31.7%) developed LRR (22.1%) and DM (17.7%). The estimated 2- and 5-year locoregional control was 78.2% (95% CI 73.3, 82.3) and 74.2% (95% CI 69.0, 78.8). One patient developed a local recurrence outside the high-dose volume and five patients developed a regional recurrence outside the high-dose volume. Four patients (1.0%) suffered a recurrence in the electively irradiated neck and two patients had a recurrence outside the electively irradiated neck. No marginal failures were observed. The estimated 2- and 5-year DM-free survival rates were 83.3% (95% CI 78.9, 86.9) and 80.0% (95% CI 75.2, 84.0). The estimated 2- and 5-year OS rates were 73.6% (95% CI 68.9, 77.8) and 52. 6% (95% CI 47.3, 57.6). Median OS was 5.5 years (95% CI 4.5, 6.7).

**Conclusion:**

Target volume definition and treatment delivery were performed accurately, as only few recurrences occurred outside the high-dose regions and no marginal failures were observed. Research on dose intensification and identification of high-risk subvolumes might decrease the risk of locoregional relapses. The results of this study may serve as reference data for comparison with future studies, such as dose escalation or proton therapy trials.

## Introduction

Head and neck cancer (HNC) is the seventh most common cancer worldwide and is usually diagnosed in a locally advanced but curable stage (1). As surgical resection can be mutilating, radiation therapy (RT), with or without concurrent chemotherapy, has emerged as the treatment of choice in the management of local and locoregionally advanced HNC (2–4). Intensity-modulated radiotherapy (IMRT) and volumetric modulated arc therapy (VMAT) are two radiation techniques that enable steeper dose gradients, allowing better sparing of the surrounding structures compared with the older 3D techniques, thereby reducing toxicity (5) and improving quality of life (6, 7). The trade-off for these more conformal RT techniques is an increased reliance on precise TV definition and accurate treatment delivery. Tumor tissue that is not defined as target volume (TV) by the radiation oncologist will not receive the prescribed dose, and geographical misses, leading to locoregional recurrences, are a potential risk (8, 9). In addition, several studies have proven experience with more conformal RT techniques to be essential for optimal outcomes in HNC (10–12). IMRT was routinely implemented in University Hospitals Leuven for the definitive treatment of HNC in 2004. Since 2010, VMAT has become the standard of care. In preparation for the implementation of proton therapy in our RT center, we investigated the quality of our current TV delineation and RT delivery by retrospectively analyzing the incidence and location of local recurrence (LR) and regional recurrence (RR) compared with the TVs. Knowledge about treatment failure patterns is especially relevant when implementing more conformal RT techniques, such as proton therapy. In the treatment of HNC, efforts are continuously being made to optimize the therapeutic ratio to improve disease outcome while keeping toxicity to a minimum. By analyzing the patterns of failure in the HNC population, we hope to refine future strategies in TV delineation and dose escalation. Moreover, the results of this study may serve as reference data for comparison with future studies, such as dose escalation or proton therapy trials.

## Materials and Methods

### Patient Selection

In this retrospective analysis, patients treated with curative intent for a HNC with R(C)T between June 2004 and December 2014 were included, to allow a follow-up of at least 3 years. We included patients with primary pharyngeal, oral cavity, and laryngeal squamous cell carcinoma. We excluded patients previously treated with RT in the head and neck region, patients with metastatic disease, postoperative patients or patients who received induction chemotherapy, primary sinonasal or nasopharyngeal tumor patients, and patients who did not finish RT as planned. The medical files were reviewed for each patient. The study was approved by the ethical committee of University Hospitals Leuven/KU Leuven (S59803). All methods were performed in accordance with the relevant guidelines and regulations.

### Target Volume Delineation and Treatment Planning

The gross tumor volume (GTV) was defined as the macroscopic tumor volume seen on planning CT and using information from clinical investigation and diagnostic and functional imaging. Patients with locally advanced disease underwent FDG-PET scan as staging exam, and PET scans were used “side-by-side” during the process of contouring with the diagnostic CT and/or MR scan. The clinical target volume of the primary tumor (CTVp) and adenopathies (CTVn) were created with a 3D 10-mm expansion around the GTV and cropped for anatomical boundaries, e.g., uninvolved bone and air. Neck regions at risk of harboring microscopic tumor cells were delineated using international guidelines (13–15) to create the elective CTV (CTVe) which received a lower dose than CTVp and CTVn. To ensure an adequate coverage of CTV, a planning target volume (PTV) was created by expanding CTV by 5 mm. The clinical target volumes of the macroscopically affected tumor sites (CTVp and CTVn) were treated up to a normalized iso-effective dose in 2 Gy fractions (NID2 Gy) of 70 Gy. CTVe was treated up to a NID2 Gy of 50 Gy, except for 19 patients that were included in a dose de-escalation trial and received a lower dose to CTVe up to a NID2 Gy of 40 Gy (16). Concurrent chemotherapy (cisplatin 100 mg/m², q3w) was offered to fit patients with advanced stage disease, according to hospital guidelines. Neck dissection post-RT was not routinely performed. Treatment plans were planned using IMRT/VMAT and tumors were classified according the American Joint Committee on Cancer seventh TNM edition (17).

### Recurrence Identification and Patient Evaluation

Recurrence and OS rates were measured starting at the start of RT until recurrence or death from any cause. CT or MRI images of the first recurrence (local/regional) were visually inspected and compared with the planning CT. Recurrences were defined as either local (LR), regional (RR), or distant metastases (DM) and further as 1) in CTVp or n, 2) marginal to CTVp or CTVn (overlap but also more than 50% of tumor load outside the original tumor site), 3) outside CTVp or CTVn, 4) outside CTVn but inside CTVe, and (5) outside CTVe. If the primary tumor or adenopathy was still visible on imaging 6 months after the start of treatment, this was classified as persistent disease (PD). Second primary (SP) HNCs were classified as such if the new tumor was more than 2 cm from the index tumor, or if it was less than 2 cm from the index tumor, but developed more than 3 years after RT.

Patients were seen 2 months after the end of therapy for a clinical evaluation which was repeated every 2 months for the first year. A CT or MRI scan was performed 4 months after the end of therapy to evaluate treatment response and once more during the first year of follow-up. Thereafter, imaging was only done in case of clinical suspicion of a recurrence. During the second, third, and fourth year, clinical follow-up was planned every 3, 4, and 6 months, respectively. Thereafter, a yearly review was planned.

### Statistical Analysis

The patients were followed up from the date of start of RT to either date of death or the cutoff date April 2018. Locoregional recurrence rates (LRR) and overall survival (OS) were estimated using the Kaplan–Meier method. The Cox proportional hazards model was used for analyzing the prognostic effect of patient or disease characteristics on oncological outcomes. Results are reported as hazard ratios with 95% confidence intervals. Univariate analysis was performed for several potential prognostic factors: age, sex, smoking status, stage, site, and tumor grade. Follow-up summary statistics were obtained using the Kaplan–Meier estimate of potential follow-up (18). Analyses were performed using SAS software (version 9.4 of the SAS System for Windows).

## Results

### Patient Characteristics

Patient characteristics of the 385 patients are shown in **Table 1**. Median age was 61 years old (range 34–89 years) and the majority were men (326 *vs.* 59 women). Seventeen patients had a multifocal tumor and two patients had an unknown primary. The primary tumor sites were the oropharynx (46.2%), hypopharynx (23.9%), supraglottis (20.8%), larynx (10.6%), and oral cavity (3.9%). Of the 178 oropharynx tumors, 39 were p16 positive, 42 were p16 negative, and 97 were of unknown status. Stage IV tumors were most common (66.5%), followed by stage III (16.9%), stage II (13.2%), and stage I (3.4%). More than half of patients were treated with concurrent chemotherapy (56.4%) and 36 were treated with an EGFR inhibitor (9.4%). Three hundred twenty-six patients were treated with accelerated RT (72 Gy in 6 weeks). Twenty-one patients were treated with adaptive RT as part of a trial and 19 patients received a lower dose to CTVe (40 Gy) in a dose de-escalation trial (16).

**Table 1:** Patient characteristics.

Patient characteristics		*n* = 385	%
Gender	Male	326	84.7%
	Female	59	15.3%
Age (years), median (range)		61	(34–89)
	<60	160	41.6%
	60–70	158	41.0%
	>70	67	17.4%
Subsite	Oropharynx	178	46.2%
	Oral cavity	15	3.9%
	Hypopharynx	92	23.9%
	Larynx	41	10.6%
	Supraglottis	80	20.8%
	CUP	2	0.5%
	Multifocal	17	4.4%
	Unifocal	368	95.6%
Grade	1	19	4.9%
	2	106	27.5%
	3	69	17.9%
	Unknown	191	49.6%
T stage	1	31	8.1%
	2	120	31.2%
	3	107	27.8%
	4	125	32.5%
	Unknown	2	0.5%
N stage	0	116	30.1%
	1	34	8.8%
	2	222	57.7%
	3	13	3.4%
Stage	1	13	3.4%
	2	51	13.2%
	3	65	16.9%
	4	256	66.5%
P16 status in oropharyngeal tumors (*n* = 178)	Negative	42	23.6%
	Positive	39	21.9%
	Unknown	97	54.5%
Smoking	Never	38	9.9%
	Former	92	23.9%
	Current	255	66.2%
Ethyl	Never	154	40.0%
	Former	85	22.1%
	Current	146	37.9%
Concomitant systemic therapy	None	132	34.3%
	Chemotherapy	217	56.4%
	EGFR inhibitor	36	9.4%
RT dose, median (range)		72 Gy	(66–75 Gy)
	66 Gy	16	4.2%
	66.1–69.99 Gy	7	1.8%

CUP, cancer of unknown primary; EGFR, epidermal growth factor.

### Survival

The median follow-up period was 6.4 years (IQR 4.7–8.3 years). The estimated 2- and 5-year OS rates were 73.6% (95% CI 68.9, 77.8) and 52.56% (95% CI 47.3, 57.6). Median OS was 5.5 years (95% CI 4.5, 6.7). The type of recurrence had a significant impact on OS; 96 of 237 patients (40.5%) with no recurrence died during follow-up *vs.* 44 of 60 patients (73.3%, RR 1.7, 95% CI 1.4, 2.1) with LRR as first recurrence and 58 of 62 patients (93.5%, RR 2.3, 95% CI 2.0, 2.7) with distant metastases. Regarding the development of a second primary in the head and neck, this had no significant impact on OS (10 of 26 patients died, 38.5%, RR 0.9, 95% CI 0.6–1.6).

### Recurrence Patterns

**Figure 1:**
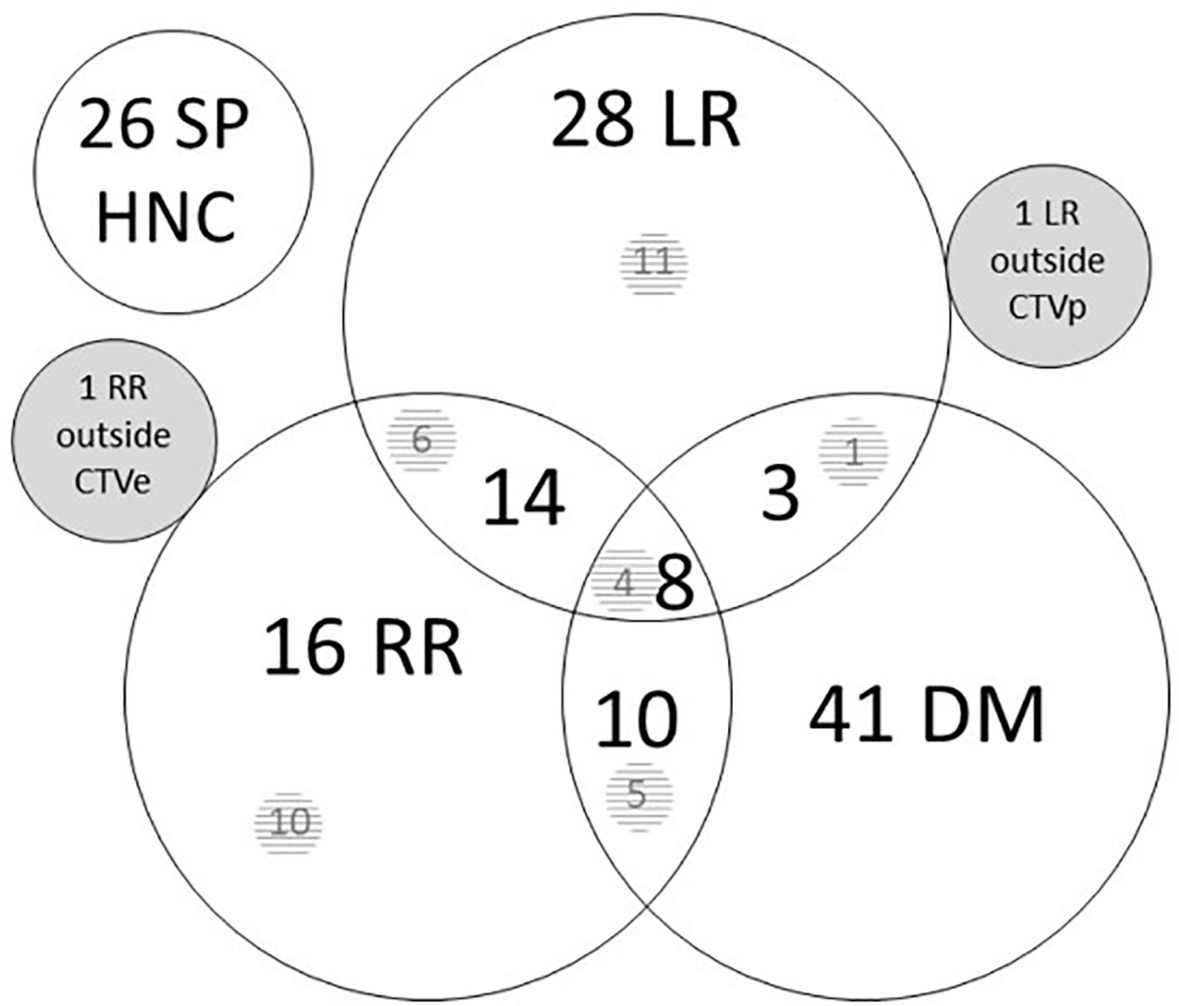
Site of first recurrence. Number of patients with a recurrence in the different sites. Overlapping circles show combination possibilities. There were one isolated local recurrence outside CTVp and one isolated regional recurrence outside CTVe. The numbers in the shaded area represent persistent tumors after the end of treatment, included in the total number of recurrences, e.g., 10 of 16 RR were persistent after treatment. CTVe, elective clinical target volume; DM, distant metastases; HNC, head and neck cancer; LR, local recurrence; RR, regional recurrence; SP, second primary.

One hundred twenty-two patients suffered a recurrence, of which the LR, RR, and DM distribution is shown in **Figure 1** for first recurrence only. Among the patients with recurrence, the median time to failure was 9.6 months (range 3.3 months to 5.5 years). Fifty-four patients (14.0%) had LR, 49 patients (12.7%) had RR, and 62 patients (16.1%) developed DM as first site of recurrence. The estimated 2- and 5-year locoregional control was 78.2% (95% CI 73.3, 82.3) and 74.2% (95% CI 69.0, 78.8). On univariate analysis, a history of ethyl abuse, a higher tumor stage, and a higher tumor grade were significantly associated with more LRR (**Table 2**). The estimated 2- and 5-year DM-free survival rates were 83.3% (95% CI 78.9, 86.9) and 80.0% (95% CI 75.2, 84.0).

**Table 2:** Univariate analysis for locoregional recurrence.

Variable	Test	Hazard ratio (95% CI)	*P*-value
Sex	Female *vs.* male	0.928 (0.514, 1.675)	0.8039
Age	+1 year	1.010 (0.987, 1.033)	0.3938
Smoking	Global test		0.3281
	Current smoker *vs.* stopped >6 m	1.462 (0.843, 2.536)	0.1764
	Current smoker *vs.* never	1.370 (0.655, 2.863)	0.4029
	Stopped >6 m *vs.* never	0.937 (0.401, 2.189)	0.8805
Ethyl	Global test		0.0064
	Stopped *vs.* yes	2.215 (1.290, 3.801)	0.0039
	Stopped *vs.* none	1.997 (1.195, 3.340)	0.0083
	Yes *vs.* none	0.902 (0.537, 1.513)	0.6956
Grade	+1 level	0.574 (0.369, 0.892)	0.0136
Highest TNM stage^a^	+1 level	1.935 (1.343, 2.788)	0.0004
Oral	Yes *vs.* no	1.888 (0.764, 4.664)	0.1683
Oropharynx	Yes *vs.* no	0.734 (0.475, 1.133)	0.1621
Glottis	Yes *vs.* no	0.939 (0.485, 1.817)	0.8513
Hypopharynx	Yes *vs.* no	1.435 (0.889, 2.316)	0.1396
Supraglottis	Yes *vs.* no	1.428 (0.885, 2.305)	0.1442

Continuous variables: HR > (<) 1 means higher (lower) risk for increasing level. Categorical variables: pairwise tests only presented if significant global P-value. Binary variables/pairwise tests: R > (<) 1 means higher (lower) risk for first category.

CI, confidence interval; m, months; TNM, tumor, node, metastasis stage.

a In case patients had multiple tumors, the tumor with the highest TNM stage was used.

### T-Site Failure

**Figure 2:**
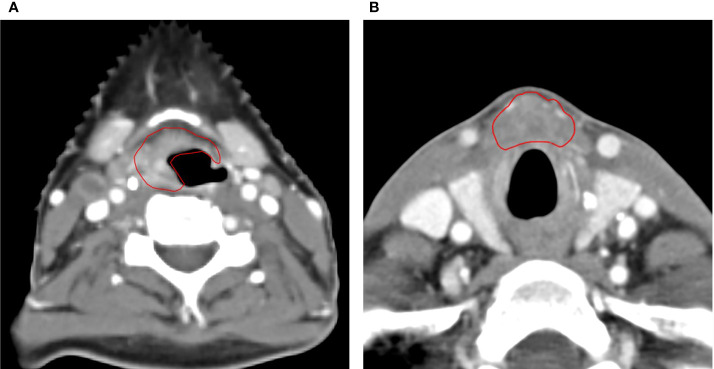
Axial CT scan images of the only patient with a local recurrence outside of the high-dose volume. **(A)** The primary tumor originating in the vallecula and **(B)** CT scan of the local recurrence in the prelaryngeal space, approximately 1 cm caudal of the index tumor.

There were 53 LRs in the high-dose volume (CTVp), no marginal recurrences, and one isolated LR outside CTVp. The latter concerned a 46-year-old female patient with two synchronous tumors: a T2 retromolar trigone tumor and a T4 tumor in the vallecula (**Figure 2A**) with multiple lymph nodes (N2b). She was treated with accelerated RT to 72 Gy concurrently with cisplatin. Ten months after the start of RT, there was a recurrence in the prelaryngeal space (**Figure 2B**) approximately 1 cm caudal of the primary tumor in the vallecula, for which she underwent a total laryngectomy. Six months later, she developed a new recurrence next to the tracheostoma for which she had re-irradiation (16 × 3.125 Gy). Half a year later, there was again local progression in combination with distant metastases. Palliative chemotherapy was started, but the patient died 6 months later.

### N-Site Failure

Of the 49 patients with RRs, 44 had recurrences inside the high-dose volume (CTVn). Four patients developed recurrences in CTVe (1.0%), of which one also had a recurrence outside CTVe in ipsilateral level V 5 years after RT. Recurrence in level II and the retropharyngeal neck level (VIIa) were the most common (level II: one ipsilateral, three contralateral; level VIIa: two ipsilateral, one contralateral). There were two RRs in level Ib (one ipsilateral and one contralateral). One patient had an isolated RR outside the irradiated volume, in the ipsilateral retropharyngeal neck 4 months after RT (**Table 3**). There were no marginal recurrences.

**Table 3:** Demographics of patients with regional recurrence outside CTVn.

No.	Sex, age, smoking	Subsite	Stage	Treatment	Failure type	Time to recurrence	Elective levels irradiated	Regional recurrence
1	Male, 46 y, former, multifocal	Piriform sinus (R) Supraglottis (L) Esophagus (mid) Oropharynx (R)	Tis T3N0 T2 Tis	70 Gy (50 Gy) + cisplatin	N+M	5 years and 2 months	Ipsi (R): Ib, II–IVa+b, Vc, VI, VIIa+b; Contra (L): II–IVa+b, Vc, VI, VIIa+b	Ipsi (R): Ib, II, V, VIIa Contra (L): Ib, II, VIIa
2	Male, 63 y, current, multifocal	1. Floor of mouth (L) 2. Tongue (R) 3. Supraglottis 4. Glottis	T2 T2N2b(R) T1 T1	72 Gy (46.4 Gy) + cisplatin	T+N+M	1 year and 6 months	Ipsi (R): Ib, II–IVa, V, VIIb Contra (L): Ib, II–IVa	Contra (L): II–III
3	Male, 67 y, former	Piriform sinus	T3N2b	72 Gy (40 Gy) + cisplatin	N+M	4 months	Ipsi: Ib, II–IVa, V, VIa+b, VIIa+b Contra: II–IVa, VIa+b, VIIa	Contra: II
4	Male, 68 y, current	Piriform sinus	T4aN2b	72 Gy (40 Gy) + cisplatin	T+N	7 months	Ipsi: II–IVa, V, VIIa+b Contra: II–III	Ipsi: VIIa-b
5	Female, 75 y, former	Piriform sinus	T2N2b	72 Gy (40 Gy)	N	4 months	Ipsi: II–IVa, V Contra: II–III	Ipsi: VIIa

Four patients had a recurrence in CTVe, of which one (patient 1) also had a recurrence outside the irradiated volume (ipsilateral level V). One patient had an isolated regional recurrence outside the elective target volume (patient 5, ipsilateral retropharyngeal adenopathy). Dose in brackets shows dose to CTVe. TNM classification according to TNM7.

Contra, contralateral; CTVe, elective clinical target volume; Ipsi, ipsilateral; L, left; N, nodal classification; R, right; T, tumor classification; y, years; M, metastasis.

### Second Primary HNC

Twenty-six patients developed SP in the head and neck region. In 8 patients, the SP developed less than 2 cm from the index tumor more than 3 years after treatment. In 3 patients, the SP was diagnosed less than 3 years after treatment of the index tumor but was more than 2 cm from the index tumor, and in 15 patients, the SP occurred more than 3 years later and more than 2 cm from the index tumor.

## Discussion

Thirty percent of HNC patients will develop a locoregional relapse, while therapy failure due to metastases is less common (19, 20). The prognosis for HNC patients after failure of first-line therapy is poor, with a median overall survival of less than 1 year (21). In order to guide future attempts to improve the therapeutic ratio and outcomes for HNC patients, understanding of the patterns of treatment failure is essential. Particularly with the introduction of IMRT and VMAT, more conformal RT techniques allowing the prescribed radiation dose to be delivered precisely to the TV, concerns about an increased risk for marginal misses were raised. Indeed, the trade-off for these more conformal RT techniques is an increased reliance on precise TV definition and accurate treatment delivery.

The present study reports the patterns of recurrence after RT in 385 HNC patients treated between 2004 and 2014 with IMRT/VMAT at the University Hospitals Leuven. Thirty-one percent of patients suffered a LRR, which corresponds with previously reported recurrence rates in HNC patients treated with definitive radio(chemo)therapy (19, 20). History of ethyl abuse and a higher TNM stage were associated with more LRR. Only one patient developed a LR outside the CTVp. As for RR, five patients suffered a recurrence outside CTVn, of which two recurrences were located outside CTVe. There were no marginal recurrences, either local or regional. Demographics of patients with a RR outside the high-dose volume are summarized in **Table 3**. Of the five regional relapses seen in our patient population, all were originally LAHNSCC and underwent PET-CT scan. The PET scans were reviewed and no missed nodes were identified. In one patient, relapse occurred in ipsilateral level V, more than 5 years after RT. It is important to note that this patient had a multifocal tumor which makes elective level selection more complicated and comes with an increased RR risk. The other patient developed a RR 4 months after the start of RT in ipsilateral level VIIa. This patient suffered from a N2b (levels III and IV) hypopharynx tumor and level VIIa was not included in the CTVe. Looking at the planning CT retrospectively, it is possible that there was a lymph node initially, although it was not withheld on FDG-PET/CT. Several trials reported a higher incidence of LRR in the lower neck (22–24). In our study, no marginal recurrences were observed, which provides reassurance about treatment quality and stresses the importance of guideline adherence for accurate neck level selection (13–15, 25). Nineteen patients were simultaneously included in a dose de-escalation trial, investigating the patterns of regional recurrences in the electively irradiated lymph node regions after dose de-escalation to 40 Gy (EQD2 Gy) (16). The inclusion criteria of this study were previously untreated, histologically proven squamous cell carcinoma of the oral cavity, oropharynx, hypopharynx, or larynx, or cervical lymph node metastases of unknown primary cancer. All macroscopically affected tumor sites (both primary tumor and affected lymph nodes) were treated up to an EQD2 Gy of 70 Gy. All 44 patients that suffered a recurrence inside CTVn were thus treated up to an EDQ2 of 70 Gy. Of the four patients that developed a RR in CTVe, none were included in the de-escalation trial, and thus, all four patients received the standard elective dose, with an EQD2 up to 50 Gy.

The vast majority of relapses, both local and regional, occurred in-field. A number of trials aimed to analyze the patterns of treatment failures after (IM)RT in HNC patients. Gujral and Nutting reviewed the data from 5 prospective randomized controlled trials, 1 prospective phase II trial, and 10 retrospective comparative series (26). Two-year locoregional control rates for IMRT fluctuated between 59% and 98.7%. Only 1 of the 16 studies reported the rates on in-field and out-of-field failures and observed more relapses in the high**-**dose region (5). Our findings are consistent with existing literature, reporting locoregional failures to predominantly occur in high-dose volumes for both the older 3D (27) and more conformal RT techniques (28–43). Compared with previous studies, our analysis provides a larger patient cohort (22, 23, 27, 31, 32, 39, 43) and longer median follow-up time (22–24, 30–32, 35, 39). Leeman et al. reported the recurrence patterns of a large cohort of 1,000 patients and found neither marginal nor out-of-field failures (29). However, heterogeneity in all reported studies renders generalization difficult. Firstly, there are differences between patient and treatment cohorts: in terms of primary histology; anatomical sites and stages (8, 19, 28, 29, 33, 34, 36–40); different types of RT intent, i.e., primary curative or adjuvant (8, 28, 34, 38); and different types of intent and uses of chemotherapy (19, 36, 38, 39, 41). Secondly, a number of studies investigate tumor persistence as part of recurrences (19, 28) or do not specify the separation at all (29, 36). The determination of out-of-field failures, defined as failure that occurred outside the treatment field, is fairly straightforward in published reports. The definition of a marginal failure is, however, not as clear-cut (22, 30, 31, 39). In the current study, we defined marginal failure as a situation in which at least one-half of the volume of the recurrence appeared to be outside the original tumor site (CTVp or CTVn) (39). Using this definition, no marginal recurrences were found, providing reassurance that TV delineation, expansion for CTV and PTV, and treatment delivery were performed adequately. By all means, we must exercise caution generalizing our results, since other IMRT series do report the occurrence of marginal failures (8, 22, 24, 27, 31, 39, 43). Furthermore, most published data are coming from single-center cohorts, provided by large-volume centers with significant experience in the treatment of HNC. This should be taken into consideration, since variations in TV delineation and treatment quality are proven to affect LRR rates. Chen et al. evaluated the pattern of RR among 107 patients who presented for consideration of re-irradiation to a large tertiary center. They found 41% of recurrences to be a marginal miss, while 18% appeared to be a true miss (8). The higher incidence of true and marginal misses in this study, compared with previously mentioned reports, could be explained by the fact that patients received their initial treatment in several lower-volume RT departments with less experience with IMRT in the HNC population. Therefore, their results might paint a more realistic picture about the recurrence patterns of HNC patients. Indeed, several studies have found a worse OS among patients treated at low-volume RT centers, with incorrect TV delineation and radiotherapy planning as the main contributors to poor outcome (11, 12). Boero et al. showed that among HNC patients treated with IMRT, for every five additional patients treated per provider per year, the risk of mortality decreased by 21% (10). These findings were backed by the RTOG 0022 study, which noted higher failure rates among patients with major protocol violations in IMRT radiation plans (43). On top of that, several studies have reported a remarkable amount of heterogeneity with respect to TV delineation among RT centers, even when delineation guidelines are available (9, 44–46). The number of marginal or out-of-field recurrences was slightly lower than observed in previous studies, except for the large cohort of MSKCC, a center with a lot of expertise in the treatment of HNC (29). Our definition of marginal failure, which was narrower compared with several other studies (30–32, 38, 39), could also have contributed to the observed results.

Nevertheless, providing accurate guideline adherence, TV definition, and treatment delivery, all reported studies confirm the predominance of in-field recurrences after IMRT. True in-field recurrences likely represent more biologically resistant tumors, which could possibly be explained by, for example, the harboring of an increased proportion of cancer stem cells and/or hypoxic elements (47, 48). Several mechanisms involved in RT response have been described in HNSCC such as hypoxia, the presence of cancer stem cells (CSC), signaling pathways, DNA damage response (DDR), and cell death pathways. It is important to keep in mind that radioresistance cannot be explained by one single mechanism or protein, but rather by an interplay of different mechanisms. Considerable evidence has suggested that tumor hypoxia results in resistance to (C)RT and tumor recurrence (48, 49), setting the stage for dose intensification strategies. A recent review concluded that dose escalation could improve OS without increased toxicity, although follow-up periods were short in small cohorts, which could result in underreporting (50). The authors concluded that functional imaging modalities could help identify the true extent of the tumor and the region that could benefit most from dose escalation, without increased toxicity. In the meantime, results of five randomized controlled trials are awaited, investigating the benefit of RT dose escalation in HNC patients (NCT01212354, NCT03376386, NCT02352792, NCT02031250, NCT03865277). Another interesting track that deserves attention is dose escalation with proton therapy, as its unique characteristics allow better sparing of normal surrounding tissue, and therefore, dose escalation is less restricted by toxicity (NCT03513042).

The strength of the current study is that it concerns a large single-institution study, in which all patients were treated in a relatively uniform manner. This way, the confounder of variation in treatment quality is minimized, which allows for the analysis of the true patterns of recurrence. There was a long follow-up period (median 6.4 years) and a large patient cohort. Our study is limited by its retrospective nature and the heterogeneity of the patient cohort. P16 status of oropharyngeal carcinoma (OPC) was unknown for 97 patients. Differences in tumor stage, tumor subsite, and the use of concomitant systemic treatment could possibly affect the pattern of failure. OPC patients are over-represented in our cohort, which follows the pattern of other large patient cohorts and is likely due to the increasing incidence of OPC. The current study is underpowered for a subgroup analysis. However, our results show a trend toward more LRR for hypopharyngeal, supraglottic, and oral cavity tumors (**Table 2**), which corresponds with the results of Leeman et al. (29). The subsites were not matched for varying stages of disease, which may affect differences in the observed outcomes. However, these variations may also more accurately reflect the clinical presentation that we deal with on a daily basis. All tumor stages [I–IV; classified according to the American Joint Committee on Cancer seventh TNM edition (17)] were included, which could affect the patterns of failure and increase the heterogeneity of the treatment. However, the primary aim of our study was to investigate the quality of current target volume delineation by analyzing the pattern of treatment failure. Since we do not adapt our CTV and PTV margins according to disease stage, we do not expect a significant impact of tumor stage on the recurrence pattern when assessing the accuracy of our GTV and CTV delineation. Another pitfall of the current study may be the cutoff period of 3 years or the cutoff distance of more than 2 cm from the index tumor, to differentiate between a LR and SP. However, this definition was based on several previous publications (30, 51).

## Conclusion

This large-institution study adds to the evidence of predominant treatment failure inside high-dose radiotherapy volumes, indicating that recurrences are mainly caused by tumor resistance. Our findings reinforce the need to focus on dose intensification and identification of high-risk subvolumes. No marginal recurrences were observed, providing reassurance about accurate TV delineation and the quality of treatment delivery. Caution must be exercised when generalizing these results, since experience with more conformal RT techniques seems a key prerequisite for favorable outcomes in the treatment of HNC patients.

## Data Availability Statement

The raw data supporting the conclusions of this article will be made available by the authors, without undue reservation.

## Ethics Statement

The studies involving human participants were reviewed and approved by the Ethics Committee Research UZ/KU Leuven. Written informed consent for participation was not required for this study in accordance with the national legislation and the institutional requirements. Written informed consent was obtained from the individual(s) for the publication of any potentially identifiable images or data included in this article.

## Author Contributions

Conceptualization: JV and SN. Data curation: JV. Formal analysis: AL. Investigation: JV. Methodology: JV. Supervision: SN. Writing—original draft: JV and HB. Writing—review and editing: HB and SN. All authors contributed to the article and approved the submitted version.

## Funding

SN is appointed as Senior Clinical Investigator by FWO—Research Foundation Flanders.

## Conflict of Interest

The authors declare that the research was conducted in the absence of any commercial or financial relationships that could be construed as a potential conflict of interest.

## Publisher’s Note

All claims expressed in this article are solely those of the authors and do not necessarily represent those of their affiliated organizations, or those of the publisher, the editors and the reviewers. Any product that may be evaluated in this article, or claim that may be made by its manufacturer, is not guaranteed or endorsed by the publisher.
